# Increased Orexin by High Concentration of Sevoflurane Enhances the Suprahyoid Muscle Activity Involves in Gasping‐Like Respiration of Mice

**DOI:** 10.1096/fba.2025-00272

**Published:** 2026-01-21

**Authors:** Yoko Irukayama‐Tomobe, Jun‐Dal Kim, Hisayo Jin, Saki Taiji, Tsuyoshi Nemoto, Dai Horiuchi, Yunosuke Ogata, Tomoya Hamamura, Tomoko Misawa, Takashi Kanbayashi, Takashi Nishino, Shiroh Isono, Takuji Suzuki, Yoshitoshi Kasuya, Koichiro Tatsumi

**Affiliations:** ^1^ Medical Mycology Research Center (MMRC) Chiba University Chiba Japan; ^2^ Department of Respirology, Graduate School of Medicine Chiba University Chiba Japan; ^3^ Division of Complex Biosystem Research, Department of Research and Development, Institute of Natural Medicine University of Toyama Toyama Japan; ^4^ AMED‐CREST, Japan Agency for Medical Research and Development Tokyo Japan; ^5^ Department of Anesthesiology, Graduate School of Medicine Chiba University Chiba Japan; ^6^ Chiba University Hospital Chiba Japan; ^7^ International Institute for Integrative Sleep Medicine (IIIS) University of Tsukuba Tsukuba Japan; ^8^ Chiba University School of Medicine Chiba Japan; ^9^ Department of Molecular and Systems Pharmacology, Faculty of Pharmacy Juntendo University Chiba Japan

**Keywords:** gasping respiration, hypothalamic transcriptome, orexin, sevoflurane, suprahyoid muscle

## Abstract

Gasping respiration enhances survival chances during cardiac arrest by activating the suprahyoid muscles (SHMs), which are crucial for airway dilation. We previously reported that the high concentration of sevoflurane (6.5%: 2.0 minimum alveolar concentration, MAC) leads to gasping‐like respiration in mice. Here, to understand the molecular mechanisms of this phenomenon, we compared the hypothalamic transcriptome profiles among control, 2.3% sevoflurane (0.7 MAC; eupnea), and 2.0 MAC groups and identified the differentially expressed genes (DEGs), in which hypocretin (orexin) precursor (*Hcrt*) gene expression was significantly elevated in the 2.0 MAC group. Notably, the intracerebroventricular administration of orexin enhanced SHM activity at 0.7 MAC. Our findings suggest that the 2.0 MAC sevoflurane‐induced increases in orexin enhance activation of SHMs resulting in the involvement of gasping respiration.

## 1 Introduction

Gasping respiration, characterized by labored breathing, is a critical self‐resuscitative mechanism during severe hypoxia. It is associated with improved survival outcomes in patients experiencing cardiac arrest [1]. The survival rate is reported to be only 9.4% in cases without gasping, whereas it rises to 39% in cases with gasping [2]. Gasping respiration has been linked to increased activity in the suprahyoid muscles (SHMs), specifically the genioglossus and geniohyoid muscles, which are crucial for tongue protrusion and maintaining upper airway patency [3]. Although gasping is thought to represent a simplified respiratory state, the precise neural and molecular mechanisms underlying this phenomenon remain elusive.

Sevoflurane, a widely used inhalational anesthetic, is favored for its minimal airway irritation, making it the agent of choice for maintaining anesthesia during spontaneous breathing [4]. Additionally, it is effective in managing severe asthma exacerbations due to its bronchodilatory and anti‐inflammatory properties [5, 6]. We have previously reported alterations in respiratory patterns with increasing sevoflurane concentrations, including increased tidal volume and decreased respiratory frequency [7]. Besides, a high concentration of sevoflurane (6.5%, 2.0 minimum alveolar concentration, MAC) induces a gasping‐like respiratory pattern, resembling that observed in mice exposed to severe hypoxia (5% O_2_) [3].

In a recent study, we found that riluzole, a persistent sodium channel blocker, suppresses both sevoflurane‐ and hypoxia‐induced gasping in mice [8]. Although their mechanisms may differ, both likely involve activation of persistent sodium currents in brainstem pacemaker neurons. We used high‐concentration sevoflurane to induce gasping because it provides a more stable and reproducible model than hypoxia‐induced gasping, which is technically more difficult to control and shows greater variability. However, our understanding of the molecular mechanisms governing gasping‐like respiration remains incomplete. In the current study, to understand the molecular mechanisms underlying the sevoflurane‐induced gasping‐like respiration in mice, we performed comprehensive hypothalamic transcriptome analysis.

## 2 Materials and Methods

### 2.1 Ethical Approval

The experimental protocols and the number of animals used in the experiments were approved by the Animal Experiments and Use Committee of the University of Chiba (approval number 6‐79). All methods were performed in accordance with the Animal Research Reporting of In vivo Experiments guidelines.

### 2.2 Experimental Setup, Measurements, and Protocols

All animal experiments were conducted as previously reported [3, 7]. Mice were tracheally intubated under 2.0%–2.5% sevoflurane anesthesia in oxygen‐enriched air and allowed to breathe spontaneously. Body temperature was maintained at 37°C ± 0.5°C using a heating pad. After confirming stable respiration and anesthesia, gasping was induced by rapidly increasing the sevoflurane concentration to 2.0 MAC (≈ 6.5%) under 100% hyperoxia, as described previously [7]. The 0.7 MAC hyperoxia condition served as the baseline control because hyperoxia alone does not induce gasping. 0.7 MAC under hyperoxic conditions was used as the baseline for comparison with 2.0 MAC (≈ 6.5%) sevoflurane. Orexin A (4 nmol, 1 μL) or vehicle was injected into the lateral ventricle via a guide cannula under sevoflurane anesthesia. Suprahyoid muscle EMG activity was recorded for 1 h after injection.

### 2.3 RNA Sequencing and Functional Enrichment Analysis

Mice were perfused intracardially with ice‐cold diethylpyrocarbonate (DEPC)‐treated PBS to remove blood cells. The cortex, cerebellum, and olfactory bulb were rapidly dissected, and the remaining hypothalamic region was homogenized in ISOGEN (Fujifilm, Japan) for total RNA extraction.

RNA sequencing analysis was performed as previously described [9]. Reads were mapped to the GRCm38/mm10 mouse reference genome and quantified using the CLC Genomics Workbench (version 23.0.5, QIAGEN). The read counts were normalized by calculating the number of reads per kilobase per million reads (RPKM) for each transcript in individual samples using CLC Genomics Workbench software (version 23.0.5, QIAGEN). The differentially expressed genes (DEGs) were identified using fold‐change −2 to +2 filtering analysis with an FDR of *p* < 0.05. The web‐based *Enrichr* suite (http://amp.pharm.mssm.edu/Enrichr) was used to assign GO terms for molecular function and biological processes enrichment of DEGs between sample groups.

### 2.4 Quantitative Real‐Time PCR Analysis

Total hypothalamic RNA from mice was reverse‐transcribed using ReverTra Ace (TOYOBO) to synthesize single‐stranded cDNA, according to the manufacturer's instructions. Quantitative real‐time PCR was performed using a duplex CFX Duet real‐time PCR system (Bio‐Rad) and SYBR Green KAPA qPCR master mix (Roche Diagnostic). The cDNA samples were amplified using predesigned primers for *Gapdh* (Primer Set ID: MA050371, Takara), *Hcrt* (forward primer, 5′‐AACTTTCCTTCTACAAAGGTTCC‐3′; reverse primer, 5′‐CGCTTTCCCAGAGTCAGGAT‐3′) in a duplex CFX Duet real‐time PCR system (Bio‐Rad). Data were normalized to the mRNA levels of *Gapdh*, and the ΔΔ*Ct* method was applied for all comparative analyses.

## 3 Results and Discussion

**FIGURE 1:**
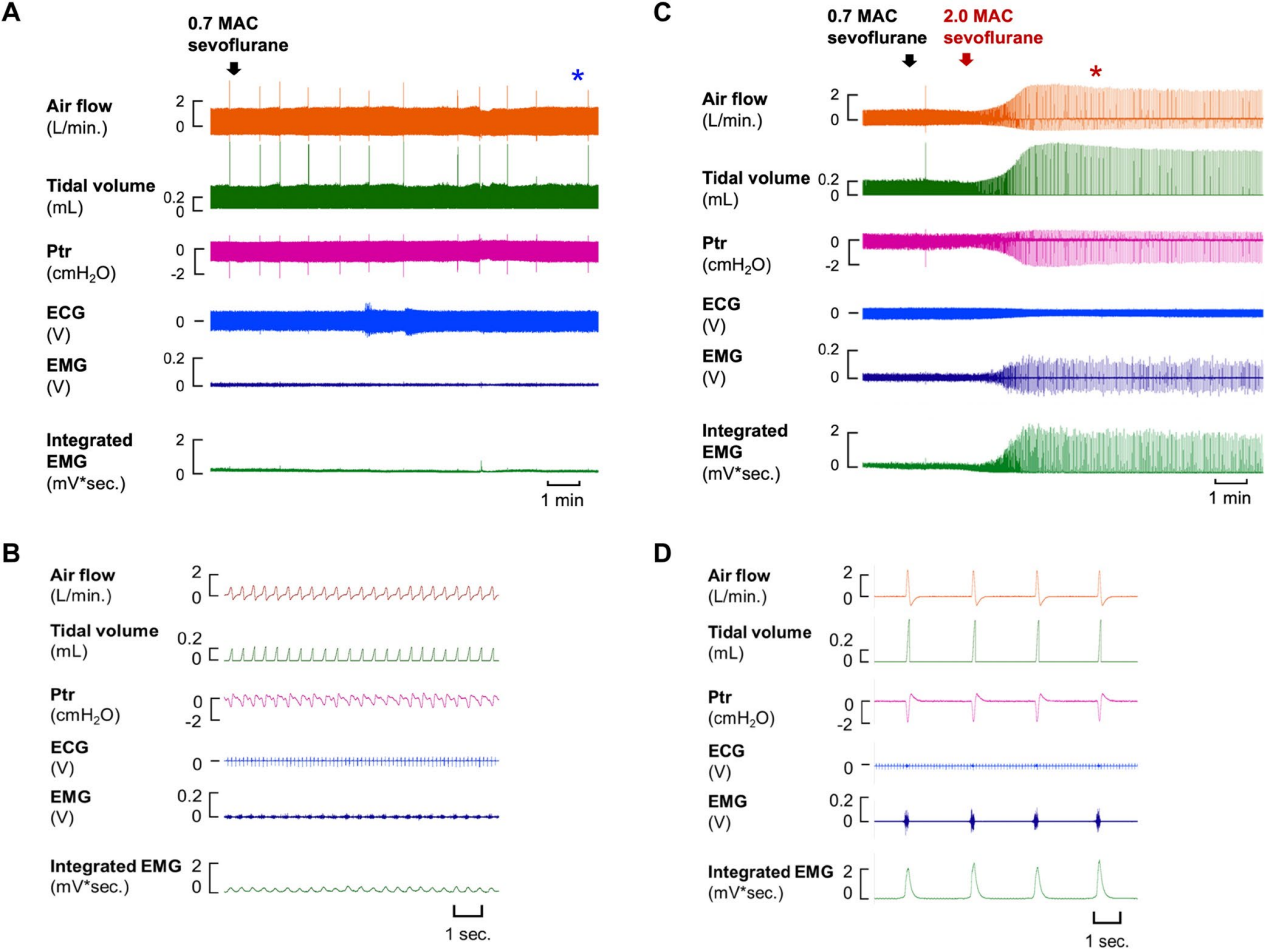
Induction of gasping‐like respiration by high concentration of sevoflurane in mice. (A) Representative traces of ventilatory responses, ECG, and EMG under eupnea (0.7 MAC sevoflurane). (B) Enlarged view of the region marked with blue asterisk in (A). (C) Representative traces of ventilatory responses, ECG, and EMG under 2.0 MAC sevoflurane. (D) Enlarged view of the region marked with red asterisk in (C).

In mice, a baseline level of sevoflurane anesthesia is maintained at 0.7 MAC. Under hyperoxic conditions, increasing the sevoflurane concentration to a deep anesthesia level (2.0 MAC) induces gasping‐like respiration, which is characterized by decreased respiratory frequency (fR) and a twofold increase in tidal volume (*V*_*t*_) compared to baseline eupnea (Figure 1A,B). At 2.0 MAC, the activity of SHMs—comprising four distinct muscles essential for mandibular movement and upper airway patency—was significantly enhanced, supporting the maintenance of gasping‐like respiration (Figure 1C,D and Table S1).

**FIGURE 2:**
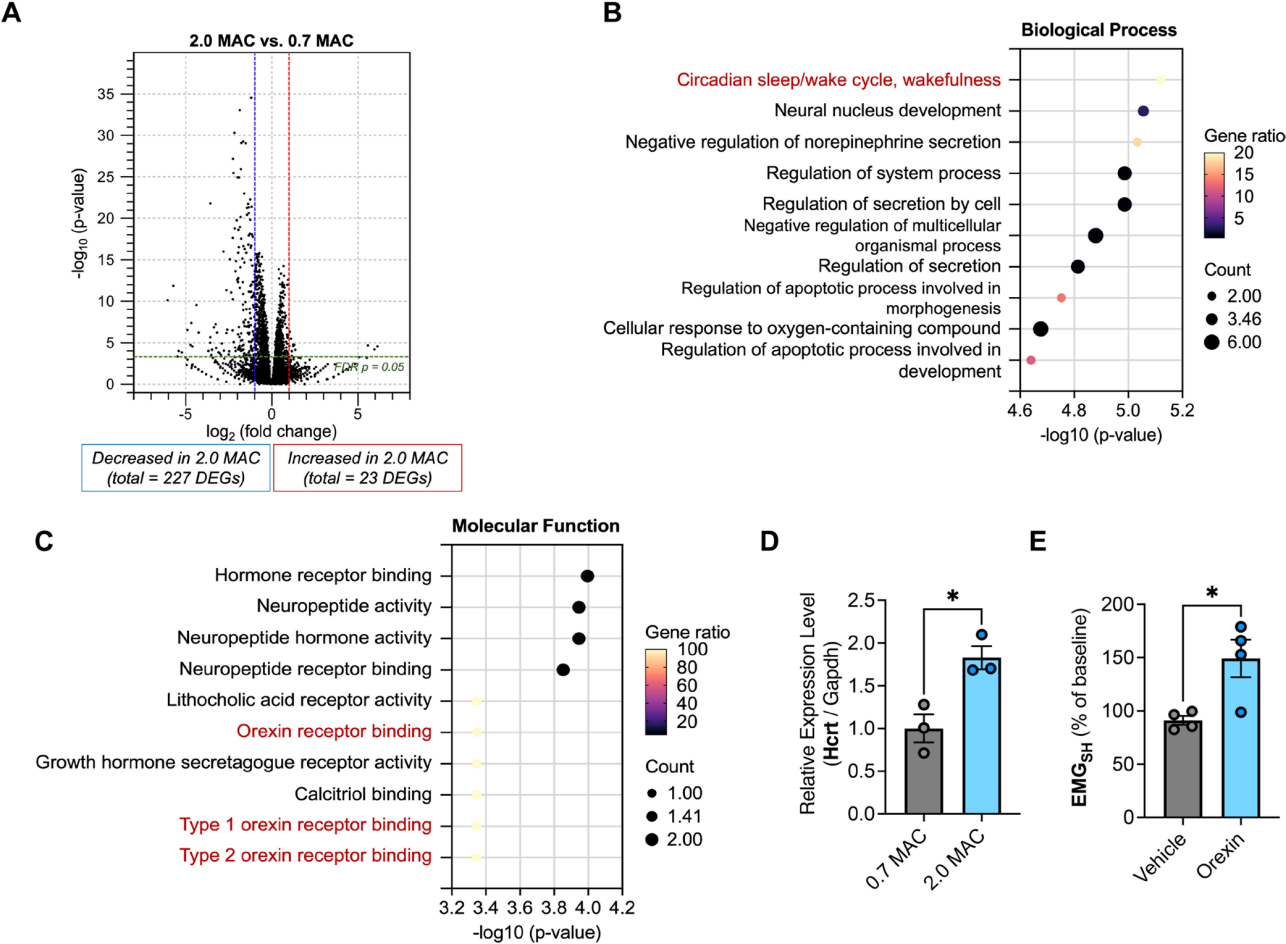
Effects of intracerebral orexin administration on the activity of suprahyoid muscle (SHM). (A) Volcano plot of DEGs comparing the 0.7 MAC and 2.0 MAC sevoflurane groups. (B, C) Gene ontology (GO) enrichment analysis (biological processes (B) and molecular functions (C)) of upregulated DEGs between two groups. (D) Expression of the *Hcrt* gene among the control group, 0.7 MAC and 2.0 MAC sevoflurane groups. The values represent the mean ± standard error of the mean (SEM) (*n* = 3 per group). Statistical analyses were conducted using unpaired two‐tailed Student's *t*‐tests, with *p < 0.05. (E) EMG activity with/without the orexin in 0.7 MAC sevoflurane groups. The values represent the mean ± SEM (*n* = 4 per group). Statistical analyses were conducted using unpaired two‐tailed Student's *t*‐tests, with *p < 0.05.

Breathing, a tightly regulated physiological process, is controlled by neural circuits that adjust respiratory drives in accordance with behavioral state and metabolic demands. Within this regulatory network, hypothalamic neurons play a pivotal role in controlling respiratory rate and depth [10]. To gain insight into how high concentrations of sevoflurane alter hypothalamic transcripts and the occurrence of gasping respiration, we conducted RNA sequencing in hypothalamic tissues of the 0.7 MAC and 2.0 MAC groups. A total of 250 DEGs were identified, of which 23 were upregulated and 227 were downregulated in the 2.0 MAC group (Figure 2A and Table S2). As shown in Figure 2B, the enriched GO terms of the upregulated 23 DEGs based on the Biological Processes showed that they were associated with “Circadian sleep/wake cycle, wakefulness” and “Regulation of secretion by cell”. Moreover, in the Molecular Function, the terms were associated with “Neuropeptide activity”, “Type 1 orexin receptor binding”, and “Type 2 orexin receptor binding” (Figure 2C). Importantly, expression of the hypocretin (orexin) neuropeptide precursor (*Hcrt*) gene, which is commonly included in the above terms, was significantly increased in 2.0 MAC mice as compared to that in 0.7 MAC mice (Figure 2D).

The hypothalamus is known as the center for defense response and modulates respiration in response to stressors [10]. Moreover, a hypothalamic neuropeptide precursor protein, orexin, is synthesized by neurons in the lateral hypothalamus and has a critical role in regulating sleep–wake cycles, energy balance, and stress responses [11]. As *Hcrt* was identified as a candidate gene associated with gasping‐like respiration, we investigated the effects of intracerebroventricular (ICV) orexin administration on respiratory parameters and SHM activity, which are essential for maintaining upper airway patency during gasping‐like respiration. To assess orexin's impact on upper airway muscle activity, we measured integrated SHM electromyographic activity (EMG_SH_). Very interestingly, ICV administration of orexin significantly increased EMG activity compared to vehicle administration under 0.7 MAC sevoflurane (Figure 2E and Table S3). These results indicate that orexin enhances SHM activity, which highlights its critical role in supporting gasping respiration under life‐threatening conditions.

It has been reported that orexin receptors are present in the hypoglossal nucleus and orexin may directly stimulate neuronal firing, promoting the activity of the genioglossus muscles, one of the dilator muscles of the airway [12]. On the other hand, orexin antagonizes the propofol, remifentanil, or sevoflurane‐induced respiratory depression [13]. Recent advancements in potent orexin agonists have demonstrated promising potential in enhancing respiratory function and mitigating opioid‐induced respiratory depression [14]. Notably, these agonists may facilitate gasping respiration, suggesting potential therapeutic applications in resuscitation efforts. Although gasping has traditionally been regarded as a prognostic marker rather than a direct therapeutic target, our findings suggest that enhancing or preserving gasping physiology could serve as an adjunctive strategy to maintain brainstem‐driven respiration and improve resuscitation outcomes when combined with conventional cardiopulmonary resuscitation and respiratory support.

## Author Contributions

Y.I.‐T., J.‐D.K., Y.K., and K.T. conceived and designed the study. Y.I.‐T., J.‐D.K., S.T., H.J., D.H., Y.K., T.N., Y.O., and T.H. performed the material preparation, data collection, and analysis. Y.I.‐T., Y.K., T.K., T.S., and K.T. provided reagents, materials, and analytical tools. Y.I.‐T., J.‐D.K., Y.K., and K.T. wrote the manuscript. All authors approved the final version of the manuscript.

## Funding

This work was supported by grants from the Japan Society for the Promotion of Science (JSPS) through the Initiative for Realizing Diversity in the Research Environment; the Ministry of Health, Labour and Welfare (MHLW), Japan, through the Intractable Respiratory Diseases and Pulmonary Hypertension Research Group (Grant Numbers 20FC1027 (to KT) and 23FC1031 (to KT)); JSPS through the Grants‐in‐Aid for Scientific Research (KAKENHI) program (Grant Numbers 19K07314, 25K11472 (to YI‐T), 24K11336 (to DH), and 24K08827 (to J‐DK)); and the Japan Agency for Medical Research and Development (AMED) through AMED‐CREST (Grant Number JP21gm1410010 (to J‐DK)).

## Conflicts of Interest

The authors declare no conflicts of interest.

## Supporting information


**Data S1:** fba270081‐sup‐0001‐Supinfo.pdf.

## Data Availability

All data required to evaluate the conclusions of the paper are presented in this study or upon reasonable request from the corresponding authors. All raw data files for the RNA‐seq analysis were deposited in the NCBI for Biotechnology Information Gene Expression Omnibus (GEO) database (https://www.ncbi.nlm.nih.gov/geo; accession number: GSE291020).
