# Supplementary material for: Increased Orexin by High Concentration of Sevoflurane Enhances the Suprahyoid Muscle Activity Involves in Gasping‐Like Respiration of Mice
Source: FASEB Bioadv. 2026 Jan 21;8(1):e70081. doi: 10.1096/fba.2025-00272 (PMC12820793; doi:10.1096/fba.2025-00272)
Supplement: Supplementary file 1 — Data S1: fba270081‐sup‐0001‐Supinfo.pdf. [file FBA2-8-e70081-s001.pdf]

Supplementary Information

Increased orexin by high concentration of sevoflurane enhances the suprahyoid muscle activity involves in gasping-like respiration of mice

Yoko Irukayama-Tomobe, Jun-Dal Kim#, Hisayo Jin, Saki Taiji, Tsuyoshi Nemoto, Dai Horiuchi, Yunosuke Ogata, Tomoya Hamamura, Tomoko Misawa, Takashi Kanbayashi, Takashi Nishino, Shiroh Isono, Takuji Suzuki, Yoshitoshi Kasuya, Koichiro Tatsumi#

#**Correspondence**: Jun-Dal Kim (jdkim@inm.u-toyama.ac.jp); Koichiro Tatsumi (tatsumi@faculty.chiba-u.jp)

Supplementary Tables S1-S3

Table S1. Effects of different sevoflurane concentrations on respiratory parameters and EMG_{SH} activity

	Baseline	0.7 MAC sevoflurane	2.0 MAC sevoflurane
**TI** (msec)	119 [115-122]	116 [115-117]	142 [136-148] $^{*\delta}$
**TE** (msec)	318 [301-323]	335 [326-346]	1810 [1644-1981] $^{*\delta}$
**fR** (bpm)	138 [135-145]	135 [130-138]	31 [28-34] $^{*\delta}$
**Vt** (ml)	0.29 [0.25-0.33]	0.31 [0.29-0.34]	0.71 [0.70-0.72] $^{*\delta}$
**Gasping Vt** (%baseline)	100	110 [103-113]	254 [215-286] *
**Vt / TI** (ml/sec)	2.44 [2.21-2.72]	2.77 [2.59-2.88]	5.11 [4.92-5.23] $^{*\delta}$
**TI / Ttot**	0.27 [0.27-0.28]	0.25 [0.25-0.26] $^{\#}$	0.07 [0.06-0.08] $^{*\delta}$
**VI** (ml/min)	40.8 [37.8-44.6]	40.7 [39.5-42.3]	21.9 [20.0-23.8] $^{*\delta}$
**Time to peak** (% TI)	65 [64-66]	67 [65-68]	52 [52-54] $^{*\delta}$
**Integrated EMGSH** (mV·sec)	0.09 [0.05-0.13]	0.08 [0.05-0.10]	0.36 [0.19-0.57]
**Gasping EMGSH** (% baseline)	100	91 [85-97]	487 [407-575] $^{\delta}$

**p* < 0.05, compared with the values during eupnea served baseline (0.7 MAC sevoflurane)
$^{\delta}$ *p* < 0.05, compared with the values during vehicle (0.7 MAC sevoflurane)
$^{\#}$ *p* < 0.05, baseline (0.7MAC sevoflurane) vs vehicle (0.7 MAC sevoflurane)
Gasping V_T and Gasping EMG_{SH} are expressed as percentages of the baseline, while the V_T and integrated EMG_{SH} value during eupnea served as the baseline.

Table S2. The expression level of identified 23 upregulated DEGs

Gene name	Fold change	FDR p-value correction	0.7 MAC_1 - RPKM	0.7 MAC_2 - RPKM	0.7 MAC_3 - RPKM	2.0 MAC_1 - RPKM	2.0 MAC_2 - RPKM	2.0 MAC_3 - RPKM
RP23-276M23.6	69.9617356	0.00149045	0	0	0	2.00321183	0.25834205	0
Gm21985	61.9919884	0.0031752	0	0	0	0	0.57500732	0
Gm13304	47.4520389	0.00117707	0	0.87702471	0	2.42311281	1.55671037	3.07950443
Gm28539	45.2359536	0.02239778	0	0	0	0.21939653	0	0.45664721
Gm9520	33.8124266	0.01901365	0	0	0	0.15664177	0.22606034	0.15525276
Vdr	4.56049857	0.03499008	0.02506267	0.01856055	0	0.06947686	0.05013338	0.10329118
Gm19345	3.17785424	0.02378517	0.2852046	0.26401585	0.40224146	0.98827864	0.74165093	0.73463638
Gm21119	2.8957572	0.02138868	0.22459014	0.6237139	0.49492685	0.85606404	1.66223369	1.00274079
Ghsr	2.54712717	0.00355512	0.08518179	0.13010806	0.06757711	0.29516827	0.2683657	0.21941317
Gm26673	2.45797153	0.00105353	0.22363689	0.60553994	0.62096099	1.04616479	1.00652747	1.20970269
Pmfbp1	2.40064563	0.03149691	0.13137033	0.07296625	0.08337582	0.34596629	0.29957242	0.13535466
Tlr2	2.39043355	0.020728	0.15660704	0.12177666	0.11595804	0.41242763	0.22555032	0.38725623
Lncenc1	2.35834257	0.0009285	0.18588842	0.23826211	0.16637699	0.45584764	0.48052717	0.5451131
Phox2b	2.34857642	0.00134239	0.10359695	0.17262074	0.23286139	0.50257155	0.40409293	0.38692865
A330033J07Rik	2.23688661	0.01573393	0.26481912	0.20003822	0.20168491	0.45514933	0.47039423	0.30559291
Prrxl1	2.22884747	0.01485663	0.14716977	0.11889694	0.15567189	0.31525192	0.33720667	0.35840593
Pitx2	2.22412466	0.00480423	0.20527909	0.12826908	0.14928253	0.53349317	0.36956165	0.29081936
Pgr15l	2.18822961	0.00027147	0.1930526	0.15596519	0.17635921	0.34056019	0.48446424	0.40685662
A230065H16Rik	2.15526986	0.03580455	0.8453975	0.54781319	0.81971706	1.75766094	1.09919309	2.25018027
Hcrt	2.15384527	0.00023285	2.45774774	1.61010897	1.19987713	4.80416893	4.61374363	2.98680201
Pax2	2.05629504	4.5338E-05	0.27924636	0.32958784	0.22153398	0.60477025	0.70171904	0.53047565
H2-T23	2.03433717	0.01957992	0.7842386	0.72597506	0.74313378	1.24552528	1.15693754	1.14470029
Crh	2.01162978	0.00136864	0.82244795	0.51639101	0.90778583	1.53647381	1.71668852	1.62109761

Table S3. Effects of orexin on respiratory parameters and EMG_{SH} under 0.7 MAC sevoflurane

	**Baseline (Vehicle)**	**Orexin icv**
**TI** (msec)	121 [120-122]	118 [108-126]
**TE** (msec)	363 [341-371]	383 [365-409]
**fR** (bpm)	124 [122-130]	119 [113-125]
**Vt** (ml)	0.30 [0.29-0.31]	0.33 [0.28-0.38]
**Gasping Vt** (%baseline)	100	111 [100-118]
**Vt / TI** (ml/sec)	2.45 [2.39-2.59]	2.87 [2.56-3.18]
**TI / Ttot**	0.25 [0.25-0.27]	0.24 [0.22-0.26]
**VI** (ml/min)	39.6 [36.1-42.6]	37.1 [32.2-44.1]
**Time to peak** (%TI)	67 [66-69]	66 [65-69]
**Integrated EMGSH** (mV･sec)	0.13 [0.10-0.16]	0.20 [0.17-0.21]
**Gasping EMGSH** (% baseline)	100	159 [139-169] *

*$p < 0.05$, compared with the values during eupnea served baseline (Vehicle). Gasping V_T and Gasping EMG_{SH} are expressed as percentages of the baseline, while the V_T and integrated EMG_{SH} value during eupnea served as the baseline.
